# Generation Scotland Teenage Recruitment – Creating, evaluating and evolving a study sign-up process in collaboration with study participants and young people

**DOI:** 10.23889/ijpds.v9i5.2861

**Published:** 2024-09-18

**Authors:** Robin Flaig, Sarah Robertson, Alex Wood, Liz Kirby, Rosie Tatham, Cathie Sudlow

**Affiliations:** 1 University of Edinburgh; 2 Health Data Research UK

## Objectives

Generation Scotland (GS) is a family-based genetic epidemiology study. Initial recruitment was ~24,000 adult volunteers from across Scotland in 2006-11 with consent for medical record linkage and re-contact. In 2022 we began recruiting another 20,000, with consent extended to administrative records, with age range now 12+.

## Methods

People join the study and complete a questionnaire online, with postal saliva sample collection for DNA. Initially, the consent process required a parent/guardian to join GS, confirm their 12-15 year-old’s capacity for enduring consent and invite their child to join. After launch, parents and young people fed back that the communication and consent methods had a negative effect on the ability of young people to join GS - impacting their autonomy, as they were unable join GS independently of their parents.

## Results

The GS Team designed a new sign-up process for 12–15 year-olds that allowed the teenager to sign up first and request that their parent agree they have capacity to consent. Creating this process was done in collaboration with the new GS YPAG. This new sign-up process was launched in September 2023. We also enabled sign-up using a mobile phone number instead of an email address.

## Conclusion

Co-design of GS recruitment with young people and parents has greatly improved the sign-up process, making GS the first UK longitudinal study where people aged 12-15 can provide enduring consent online with parental confirmation of their capacity. This provides young people with greater ownership over their ability to take part in health research.

